# Light induces changes in activities of Na^+^/K^+^-ATPase, H^+^/K^+^-ATPase and glutamine synthetase in tissues involved directly or indirectly in light-enhanced calcification in the giant clam, *Tridacna squamosa*

**DOI:** 10.3389/fphys.2015.00068

**Published:** 2015-03-06

**Authors:** Yuen K. Ip, Biyun Ching, Kum C. Hiong, Celine Y. L. Choo, Mel V. Boo, Wai P. Wong, Shit F. Chew

**Affiliations:** ^1^Department of Biological Sciences, National University of Singapore, SingaporeSingapore; ^2^The Tropical Marine Science Institute, National University of Singapore, SingaporeSingapore; ^3^Natural Sciences and Science Education, National Institute of Education, Nanyang Technological University, SingaporeSingapore

**Keywords:** ammonia, Ca^2+^-ATPase, H^+^-ATPase, mantle, tridacnid, zooxanthellae

## Abstract

The objective of this study was to determine the effects of 12 h of exposure to light, as compared with 12 h of exposure to darkness (control), on enzymatic activities of transporters involved in the transport of NH^+^_4_ or H^+^, and activities of enzymes involved in converting NH^+^_4_ to glutamate/glutamine in inner mantle, outer mantle, and ctenidia of the giant clam, *Tridacna squamosa*. Exposure to light resulted in a significant increase in the effectiveness of NH^+^_4_ in substitution for K^+^ to activate Na^+^/K^+^-ATPase (NKA), manifested as a significant increase in the Na^+^/NH^+^_4_-activated-NKA activity in the inner mantle. However, similar phenomena were not observed in the extensible outer mantle, which contained abundant symbiotic zooxanthellae. Hence, during light-enhanced calcification, H^+^ released from CaCO_3_ deposition could react with NH_3_ to form NH^+^_4_ in the extrapallial fluid, and NH^+^_4_ could probably be transported into the shell-facing inner mantle epithelium through NKA. Light also induced an increase in the activity of glutamine synthetase, which converts NH^+^_4_ and glutamate to glutamine, in the inner mantle. Taken together, these results explained observations reported elsewhere that light induced a significant increase in pH and a significant decrease in ammonia concentration in the extrapallial fluid, as well as a significant increase in the glutamine concentration in the inner mantle, of *T. squamosa*. Exposure of *T. squamosa* to light also led to a significant decrease in the N-ethylmaleimide (NEM)-sensitive-V-H^+^-ATPase (VATPase) in the inner mantle, and significant increases in the Na^+^/K^+^-activated-NKA, H^+^/NH^+^_4_-activated-H^+^/K^+^-ATPase, and NEM-sensitive-VATPase activities in ctenidia, indicating that light-enhanced calcification might perturb Na^+^ homeostasis and acid/base balance in the hemolymph, and might involve the active uptake of NH^+^_4_ from the environment. This is the first report on light having direct enhancing effects on activities of certain transporters/enzymes related to light-enhanced calcification in the inner mantle and ctenidia of *T. squamosa*.

## Introduction

Giant clams of the family Tridacnidae are familiar and conspicuous residents of shallow coral reefs throughout the tropical Indo-Pacific (Rosewater, 1965). Like reef-building corals and anemones which harbor symbiotic dinoflagellates, these giant clams live in symbiosis with mircroalgae (*Symbiodinium* spp.), commonly known as zooxanthellae. The zooxanthellae live extracellularly in a branched tubular system originating from the stomach of the giant clam, which splits into small and very thin secondary and tertiary tubes dorsally into the root of the siphonal mantle. The tertiary tubes are directly under the surface of the extensible mantle tissue (Norton et al., 1992), allowing sufficient light to penetrate for algal photosynthesis. With this alga-invertebrate association, giant clams are able to maintain high growth rates and productivity in nutrient deficient marine environments. Thus, the availability of light is a critical factor affecting the growth of giant clams (Lucas et al., 1989).

Kawaguti and Sakumoto (1948) reported that scleractinian corals calcified faster in light than in darkness, a phenomenon which was confirmed later by Goreau (1959) and subsequently called light-enhanced calcification. In general, four hypotheses have been proposed for light-enhanced calcification in corals and they may also apply to *Tridacna* spp. Firstly, the production of H^+^ by calcification favors CO_2_ formation (Goreau, 1959), and photosynthetic activity of the symbiotic dinoflagellates lowers the CO_2_ partial pressure in the calcification site and thus favors calcium carbonate precipitation (McConnaughey and Whelan, 1997). Secondly, light-enhanced calcification can be facilitated by the removal of inhibiting substances, such as phosphates by the symbiotic dinoflagellates during photosynthesis (Simkiss, 1964). Thirdly, calcification can be enhanced through the production and release of photosynthetic products (Chalker and Taylor, 1975) and/or O_2_ (Rinkevich and Loya, 1984) by symbionts in the presence of light. In the case of tridacnids, zooxanthellae are known to translocate photosynthates to the host (Streamer et al., 1988). Fourthly, light can enhance calcification through the increase in supply of organic precursors that are needed for organic matrix synthesis from the symbionts (Muscatine and Cernichiari, 1969) to the calicoblastic cells of the animal host (Puverel et al., 2005).

Separately, Campbell and Speeg (1969) proposed that calcification in molluscs could also be enhanced by the removal of H^+^ by NH_3_ produced through the action of urease on urea in molluscs in general. In this report, ammonia refers to both NH_3_ and NH^+^_4_, while NH_3_ and NH^+^_4_ represent unionized molecular ammonia and ammonium ion, respectively. NH_3_ released through deamination of purine, purine nucleotides, and purine nucleosides could also be involved in enhancing the calcification rate (Campbell and Boyan, 1976; Loest, 1979). From the activities of adenosine deaminase and urease in 14 mollusc species examined, Loest (1979) calculated that the rate of NH_3_ formation was more than adequate to react with H^+^ released with shell growth in molluscs. Opposing that proposition, Simkiss (1976) opined that NH_3_ production was a poor mechanism for removing H^+^ because the NH^+^_4_ formed would have difficulties in permeating biomembranes and would therefore accumulate in the extrapallial fluid. However, Simkiss (1976), at that time, was unaware of channels that could facilitate NH_3_/NH^+^_4_ permeation or transporters that could actively transport NH^+^_4_.

The major nitrogenous excretory product in bivalves is ammonia (Bishop et al., 1983). In aqueous solution, ammonia exists as NH_3_ and NH^+^_4_, and the equilibrium reaction can be written as NH_3_ + H_3_O^+^ ⇔ NH^+^_4_ + H_2_O. As the pK of this reaction is around 9.0–9.5, NH_3_ acts as a base and binds with H^+^ to form NH^+^_4_ in water at neutral pH. Hence, at physiological pH, ~99% of ammonia is present as NH^+^_4_. In bivalves, NH_3_ and NH^+^_4_ participate in acid-base balance in extracellular fluids and restrict the rate of shell decalcification during hypoxia (Bayne et al., 1976; Shick et al., 1988). However, there is a dearth of knowledge on mechanisms involved in NH_3_/NH^+^_4_ transport between the extrapallial fluid and the inner mantle of bivalves. In the past, it was assumed that ammonia permeated biomembranes mainly as molecular NH_3_, and biomembranes had low permeability to the cationic NH^+^_4_. To date, NH_3_/NH^+^_4_ transport in animals is known to be facilitated by several channel proteins and transporters, including aquaporin (Holm et al., 2005; Ip et al., 2013b), Rhesus glycoprotein (Nakada et al., 2007), Na^+^/H^+^ exchanger (NHE; Good, 1994), Na^+^:K^+^:2Cl^−^ cotransporter (NKCC; Good, 1994; Loong et al., 2012; Ip et al., 2013a) Na^+^/K^+^-ATPase (NKA; Mallery, 1983) and H^+^/K^+^-ATPase (HKA; Swarts et al., 2005). NH^+^_4_ can substitute for H^+^ to activate NHE, or substitute for K^+^ to activate NKCC, NKA, and HKA.

Contrary to Simkiss's (1976) opinion, Ip et al. (2006) demonstrated that the infusion of NH_4_Cl into the extrapallial fluid of *Tridacna squamosa* led to an instantaneous increase in the total ammonia concentration therein, but the total ammonia concentration decreased subsequently and returned to the control level within 1 h. This is indicative of NH_3_/NH^+^_4_ being transported from the extrapallial fluid to the inner mantle. Additionally, they reported that the infusion of HCl into the extrapallial fluid led to an instantaneous decrease in the pH of the extrapallial fluid, but the pH increased significantly within 1 h and achieved a partial recovery toward the control pH value (Ip et al., 2006). During this 1-h period, the increase in pH was accompanied by a significant decrease in the total ammonia concentration in the extrapallial fluid, which supports the proposition that H^+^ can combine with NH_3_ and be transported as NH^+^_4_ into the tissues of the inner mantle. More importantly, Ip et al. (2006) reported that exposure of *T. squamosa* to light for 12 h induced a significant increase in the pH of, and a significant decrease in the total ammonia concentration in, the extrapallial fluid (Ip et al., 2006). There was also a significant increase in the glutamine concentration in the inner mantle of giant clams exposed to light. Thus, Ip et al. (2006) proposed that light-enhanced calcification in *T. squamosa* was achieved through the transport of H^+^ as NH^+^_4_ from the extrapallial fluid to the inner mantle tissues, where NH^+^_4_ could act perhaps partially as a substrate for glutamine production.

Therefore, the first objective of this study was to determine, at saturating substrate concentrations and optimal assay conditions, the mass-specific enzymatic activities of Ca^2+^-ATPase, Mg^2+^-ATPase, NKA (activated by Na^+^/K^+^ or Na^+^/NH^+^_4_ and inhibited by ouabain), HKA (activated by H^+^/K^+^ or H^+^/NH^+^_4_ and inhibited by oligomycin) and V-type H^+^-ATPase (VATPase; inhibited by bafilomycin or N-ethylmaleimide) from the inner mantle, which was adjacent to the extrapallial fluid and inside the pallial line, of *T. squamosa* exposed to 12 h of darkness (control) or 12 h of light. For comparison, we also evaluated the effects of light on the activities of these transporters from the extensible outer mantle, which was outside the pallial line, and ctenidia, which do not participate directly in the calcification process. The hypothesis tested was that the inner mantle of *T. squamosa* possessed transporters which could catalyze the active transport of NH^+^_4_, and that light could induce increases in the mass-specific activities of some of these transporters. As Mg^2+^-ATPase is not directly involved in the calcification process, its activity should not be affected by light and could therefore act as a light-refractory reference. The second objective was to examine the effects of 12 h of light exposure on the mass-specific activity of glutamine synthetase (GS), which catalyzes the formation of glutamine from NH^+^_4_ and glutamate (Walsh and Henry, 1991), and glutaminase (GA), which catalyzes the breakdown of glutamine to NH_3_ and glutamate (Walsh and Henry, 1991), in the inner mantle, the outer mantle and ctenidia. When operating in conjunction with each other, GS and GA theoretically constitute a glutamine-glutamate cycle that can turn NH^+^_4_ to NH_3_. As glutamate can in turn be formed from NH^+^_4_ and α-ketoglutarate catalyzed by glutamate dehydrogenase (GDH), efforts were also made to determine the effect of light exposure on the amination and deamination activities of GDH in these three tissues/organs. The hypothesis tested was that the inner mantle of *T. squamosa* possessed all three enzymes and that the mass-specific activities of GS in the inner mantle would increase significantly in response to light.

## Materials and methods

### Animals

Specimens of *T. squamosa* (540 ± 221 g; mean ± S.D.) were obtained from Iwarna Aquafarm Pte Ltd. (Singapore), and kept in an indoor aquarium. Giant clams (*N* = 8) were maintained in 350 l of recirculating seawater in a glass tank (L90 × W62 × H60 cm) under a 12 h light:12 h dark regime. The conditions of the water were as follow: temperature, 23–24°C; salinity, 30–32; pH, 8.1–8.3; hardness, 143–179 ppm; calcium, 280–400 ppm, phosphate, <0.25 ppm, nitrate, 0 ppm; total ammonia, <0.25 ppm. The tank was illuminated with two sets of Aquazonic T5 lighting systems (Yi Hu Fish Farm Trading, Singapore), each with four 39 W fluorescence tubes (90 cm; 2× Sun tubes and 2× Actinic blue tubes), from the top. The shaded light intensity measured at the depth of the giant clams under water using a Light Meter Lux/FC, model no 840020 (SPER Scientific Inc., USA) was 6000 LUX or 81 PPF (μmol m^−2^ s^−1^). Giant clams were acclimatized to laboratory conditions for 1 month before experiments.

### Experimental conditions and tissue collection

A batch of giant clams (*N* = 4; control) were sampled at the end of the 12 h dark period of the 12 h light:12 h dark regime. Another batch of giant clams (*N* = 4) were sampled 12 h later after exposure to light. For tissue sampling, giant clams were forced open, and the abductor muscles were cut. Samples of the inner mantle were dissected from mantle tissues adjacent to the extrapallial fluid within the pallial line, while those of the outer mantle were excised from the extensible middle and inner folds of the mantle outside the pallial line. Samples of ctenidia were also dissected out. All samples were blotted dry and immediately freeze-clamped with liquid-nitrogen-precooled aluminum tongs. Separate sets of tissues were removed for assaying of ATPase activities; they were suspended in 1 ml of solution containing 100 mmol l^−1^ imidazole-HCl buffer (pH 7.1), 300 mmol l^−1^ sucrose, 20 mmol l^−1^ EDTA following the method of Zaugg (1982). All samples were stored at −80°C until analyzed.

### Determination of chlorophyll concentration

Chlorophylls a and c2 were extracted by incubating tissue samples in 1.5 ml of cold acetone for 24 h at 4°C in the dark, and quantified spectrophotometrically at 630 and 663 nm, respectively, according to Jeffery and Humphrey (1975). The chlorophyll concentration is expressed as μg g^−1^ wet mass tissue.

### Determination of mass-specific activities of various ATPases

The tissue sample collected according to Zaugg (1982) was thawed on ice and washed 3 times with a washing buffer containing 100 mmol l^−1^ imidazole-HCl buffer (pH 7.1) and 300 mmol l^−1^ sucrose. The sample was then blotted dry, re-suspended in 5 volume (v/m) of homogenizing buffer containing 100 mmol l^−1^ imidazole-HCl buffer (pH 7.1), 300 mmol l^−1^ sucrose and 0.1% of sodium deoxycholate, and homogenized at 13,500 rpm using a Polytron homogenizer for 15 s first followed by 10 s twice, with an interval of 10 s between homogenizations. The homogenized sample was centrifuged at 4,000 ×*g* at 4°C for 6 min and the supernatant obtained was used for determining the activities of various ATPases. The protein content of the supernatant was determined by the method of Bradford (1976), with bovine γ-globulin dissolved in 25% glycerol as a standard for comparison.

For ATPase assays in general, the procedure of Ip et al. (1991, 2012a) and Chew et al. (2014) were followed. The sample was pre-incubated for 10 min at 25°C in 1 ml of reaction mixture containing 0.05 ml of sample (~0.1 mg protein), 30 mmol l^−1^ imidazole-HCl buffer (pH 7.1), with or without the prescribed substrates and/or inhibitor (as stated below), before the addition of 50 μl of 3.5 mmol l^−1^ ATP. Preliminary experiments were performed to optimize the substrate concentrations used for various enzyme assays. At saturating substrate concentrations, the enzyme activity obtained would be close to the theoretical *V*_max_, and reflect on the total concentration of enzyme present. The activity of Mg^2+^-ATPase was determined in the presence of 5 mmol l^−1^ MgCl_2_; a blank was performed in the absence of MgCl_2_ (Lin and Way, 1984). The activity of Ca^2+^-ATPase was determined in the presence of 5 mmol l^−1^ CaCl_2_; a blank was performed in the absence of CaCl_2_. The activity of Na^+^/K^+^-activated-NKA was determined in the presence of 120 mmol l^−1^ NaCl, 20 mmol l^−1^ KCl and 5 mmol l^−1^ MgCl_2_, and calculated as a difference of activities assayed in the presence and absence of 2 mmol l^−1^ ouabain. To assay for Na^+^/NH^+^_4_-activated-NKA activity, 20 mmol l^−1^ NH_4_Cl was used as a substrate instead of KCl, and a blank was determined in the presence of 2 mmol l^−1^ ouabain. The activitis of non-gastric H^+^/K^+^-activated-HKA and H^+^/NH^+^_4_-activated-HKA were determined in the presence of 20 mmol l^−1^ KCl and 20 mmol l^−1^ NH_4_Cl, respectively, together with 5 mmol l^−1^ MgCl_2_, 2 mmol l^−1^ ouabain, 5 mM NaN_3_, and 20 μl ethanol. Blanks were determined in the presence of 50 μmol l^−1^ oligomycin (dissolved in 20 μl ethanol). Preliminary experiment using SCH28080 as an inhibitor indicated the absence of gastric HKA activity in the three tissues/organ of *T. squamosa*. The activity of bafilomycin-sensitive-VATPase was assayed in the presence of 5 mmol l^−1^ MgCl_2_, 2 mmol l^−1^ ouabain and 5 mmol l^−1^ NaN_3_ and 20 μl of dimethylsulfoxide, and a blank was performed in the presence of a final concentration of 30 μmol l^−1^ bafilomycin (dissolved in 20 μl of dimethylsulfoxide). The activity of N-ethylmaleimide (NEM)-sensitive-VATPase was assayed in the presence of 5 mmol l^−1^ MgCl_2_, 2 mmol l^−1^ ouabain and 5 mmol l^−1^ NaN_3_ and 20 μl of ethanol, and a blank was performed in the presence of a final concentration of 1 mM NEM (dissolved in 20 μl of ethanol).

After the addition of 50 μl of 3.5 mmol l^−1^ ATP, the reaction mixture was incubated at 25°C for 40 min. Preliminary studies revealed that at the prescribed sample protein concentration, the reaction rates of various types of ATPases were linear up to 60 min. At the end of the incubation period, 0.05 ml of ice-cold 100% trichloroacetic acid was added to stop the reaction, and the resulting mixture was centrifuged at 10,000 ×*g* and 4°C for 2 min. The ATPase activity was measured as the amount of inorganic phosphate (Pi) released from ATP during the incubation period. For Pi assay, an aliquot (0.4 ml) of the supernatant was diluted with 4 volumes of 0.1 mol l^−1^ sodium acetate. To this, 0.2 ml of 1% ascorbic acid and 0.2 ml of 1% ammonium molydate in 0.05 mol l^−1^ H_2_SO_4_ were added. Absorbance was determined at 700 nm using a Shimadzu (Kyoto, Japan) UV160 UV-VIS spectrophotometer, and phosphate concentration calculated with reference to a standard made from KH_2_PO_4_. The ATPase activity is expressed as μmol Pi released min^−1^ g^−1^ wet mass tissue.

### Determination of specific activities of GS, GA, and GDH

The frozen samples of the inner mantle, the outer mantle and ctenidia were weighed, ground to a powder in liquid nitrogen and homogenized, using an Ika-werk Staufen Ultra-Turrax homogenizer, 3 times in 5 volumes (w/v) of ice-cold extraction buffer containing 50 mmol l^−1^ imidazole-HCl (pH 7.0), 50 mmol l^−1^ NaF, 3 mmol l^−1^ EGTA, 3 mmol l^−1^ EDTA at 24,000 rpm for 20 s each with intervals of 10 s between each homogenization. The homogenate was centrifuged at 10,000 ×*g* at 4°C for 20 min to obtain the supernatant. The supernatant obtained was passed through a 5 ml Econo-Pac 10DG desalting column (Bio-Rad Laboratories Inc., CA, USA) equilibrated with 50 mmol l^−1^ imidazole-HCl (pH 7.0). The resulting eluent was used for enzyme assays. Protein concentrations of the extract were monitored before and after gel filtration to determine the dilution factor involved.

The GS transferase activity was determined by the method of Shankar and Anderson (1985) with some modifications. Of note, the synthetase activity of GS present in the mantle of *T. squamosa* was too low to be assayed, and the transferase activity is known to be ~20-fold greater than the synthetase activity. The reaction mixture consisted of 67 mmol l^−1^ imidazole (pH 7.0), 25 mmol l^−1^ hydroxylamine, 30 mmol l^−1^ arsenate, 100 mmol l^−1^ glutamine, 1 mmol l^−1^ ADP, 1.7 mmol l^−1^ MnCl_2_, and 0.1 ml sample in a final volume of 0.8 ml. After incubation at 25°C for 10 min, the reaction was terminated with 0.4 ml of ferric chloride reagent (0.37 mmol l^−1^ FeCl_3_, 0.20 mmol l^−1^ TCA, and 0.67 mmol l^−1^ HCl). The solution was centrifuged at 10,000 ×*g* at 4°C for 5 min to remove precipitated proteins. Reaction terminated at time zero was used as blank. GS activity was measured at 500 nm and expressed as μmol γ-glutamyl hydroxamate formed min^−1^ g^−1^ wet mass tissue. Freshly prepared glutamic acid γ-monohydroxamate solution was used as a standard for comparison.

The GA activity was assayed according to the method of Curthoys and Lowry (1973). For phosphate-independent GA, the reaction mixture comprised 60 mmol l^−1^ maleate buffer (pH 6.6), 0.2 mmol l^−1^ EDTA, 10 mmol l^−1^ L-glutamine and 0.1 ml of sample in a total volume of 1.5 ml. The reaction mixture for phosphate-dependent GA in a total volume of 1.5 ml consisted of 50 mmol l^−1^ Tris buffer (pH 8.6), 150 mmol l^−1^ K_2_HPO_4_, 0.2 mmol l^−1^ EDTA, 25 mmol l^−1^ L-glutamine and 0.1 ml of sample. The reaction mixture was incubated at 25°C for 30 min, and the reaction was stopped by adding 1 M (for phosphate-independent GA) or 3 M HCl (for phosphate-dependent GA). Reaction stopped at time zero constituted the blank. The reaction mixture was centrifuged at 10,000 ×*g* at 4°C for 5 min to remove precipitated proteins. An aliquot of this reaction mixture was then added to 65 mmol l^−1^ TEA-phosphate buffer (pH 8.6), 0.5 mmol l^−1^ NAD, 0.08 mmol l^−1^ INT, 3 U of diaphorase and 14 U of GDH in a final volume of 1.5 ml. The amount of glutamate formed was determined at 492 nm using L-glutamate as a standard. Glutaminase activity was expressed as μmol glutamate formed min^−1^ g^−1^ wet mass tissue.

The GDH activity was assayed in the amination direction according to the method of Male and Storey (1983) with some modifications. The assay was performed in a total volume of 1.0 ml containing 50 mmol l^−1^ imidazole (pH 7.0), 300 mmol l^−1^ ammonium acetate, 5 mmol l^−1^ α- ketoglutarate and 0.10 mmol l^−1^ NADPH. Change in absorbance was monitored at 340 nm and expressed as μmol NADPH oxidized min^−1^ g^−1^ wet mass tissue. For the deamination reaction, sample was added to a reaction mixture containing 50 mmol l^−1^ Tris-HCl buffer (pH 8.5), 30 mmol l^−1^ L-glutamate and 0.25 mmol l^−1^ NADP. The formation of NADPH was monitored at 340 nm and enzyme activity was expressed as μmol NADPH formed min^−1^ g^−1^ wet mass tissue.

### Statistical analysis

Results are presented as means ± standard errors of means (S.E.M.). Statistical analyses were performed using SPSS version 21 (IBM Corporation, Armonk, NY, USA). Homogeneity of variance was checked using Levene's Test. Differences among means of the three tissues/organ in light or in darkness were evaluated by One-Way analysis of variance (ANOVA) followed by multiple comparisons of means by Dunnett's T3 (for unequal variance) or by Tukey's test (for equal variance). Differences between means of the same tissue/organ in light and in darkness were evaluated by Student's *t*-test. Differences were regarded as statistically significant at *P* < 0.05.

## Results

### Concentrations of chlorophyll and water soluble proteins

The inner mantle of *T. squamosa* exposed to 12 h of darkness contained a significantly lower (~20%) concentration (0.87 ± 0.06 μg g^−1^ wet mass tissue; *N* = 4) of chlorophyll than the outer mantle (4.31 ± 0.33 μg g^−1^ wet mass tissue; *N* = 4), but chlorophyll was undetectable in the ctenidia. Exposure to light for 12 h had no significant effects on the concentrations of chlorophyll in the inner mantle (0.81 ± 0.05 μg g^−1^ wet mass tissue; *N* = 4) and the outer mantle (4.35 ± 0.56 μg g^−1^ wet mass tissue; *N* = 4) of *T. squamosa*.

In addition, the inner mantle of giant clams exposed to 12 h of darkness had significantly lower water-soluble protein concentration (1.64 ± 0.80 μg g^−1^ wet mass tissue) than those of the outer mantle (4.75 ± 0.20 μg g^−1^ wet mass tissue) and ctenidia (3.95 ± 0.67 μg g^−1^ wet mass tissue), and the water-soluble protein concentrations in these three tissues/organ remained unchanged after 12 h of light exposure. Due to the variation in water-soluble protein concentrations in samples from the three tissues/organ, enzyme activities were calculated with reference to the tissue wet mass instead of the tissue protein concentration to achieve a valid comparison.

### Activities of Ca^2+^-ATPase and Mg^2+^-ATPase

**Figure 1:**
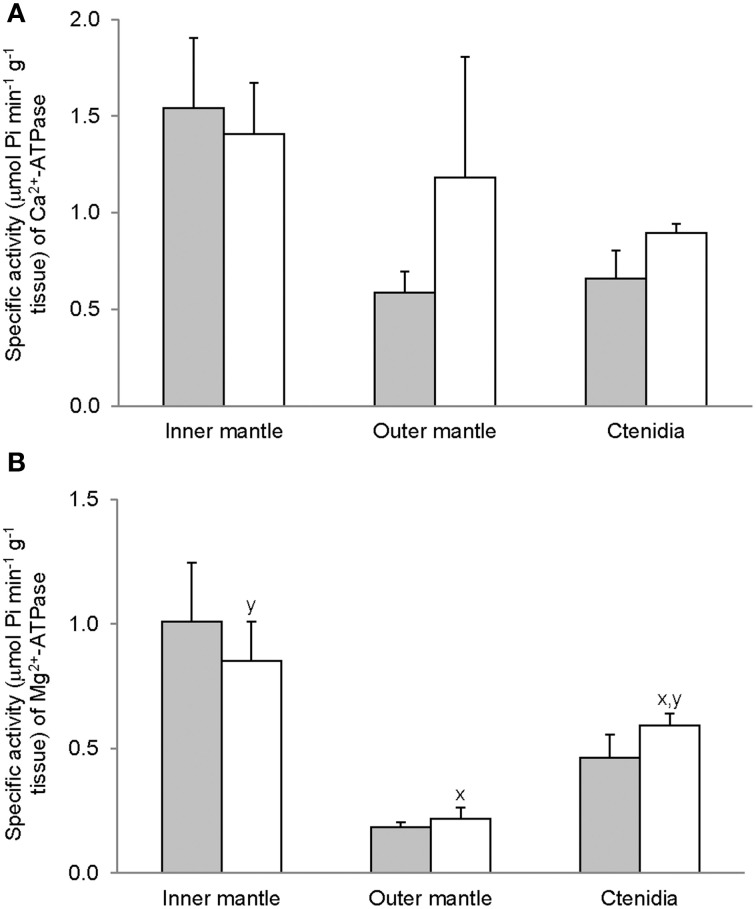
**Mass-specific activity (μmol Pi min^−1^ g^−1^ tissue) of **(A)** Mg^2+^-ATPase and **(B)** Ca^2+^-ATPase from the inner mantle, the outer mantle, and ctenidia of *Tridacna squamosa* exposed to 12 h of darkness (control; closed bar) or 12 h of light (open bar)**. Values represent means + S.E.M. (*N* = 4). Means not sharing the same letter (x, y) are significantly different from each other (*P* < 0.05).

As hypothesized, Mg^2+^-ATPase activities from the inner mantle, the outer mantle and ctenidia of *T. squamosa* were unaffected by 12 h of light exposure (Figure 1A). Activities of Ca^2+-ATPase^ were higher in the inner mantle than in the outer mantle of *T. squamosa* exposed to 12 h of light (Figure 1B). Similar to Mg^2+^-ATPase, exposure to light for 12 h had no significant effects on Ca^2+^-ATPase activity from the inner mantle, the outer mantle, and ctenidia (Figure 1B).

### Activities of ouabain-sensitive Na^+^/K^+^-activated-NKA and Na^+^/NH^+^_4_-activated-NKA

**Figure 2:**
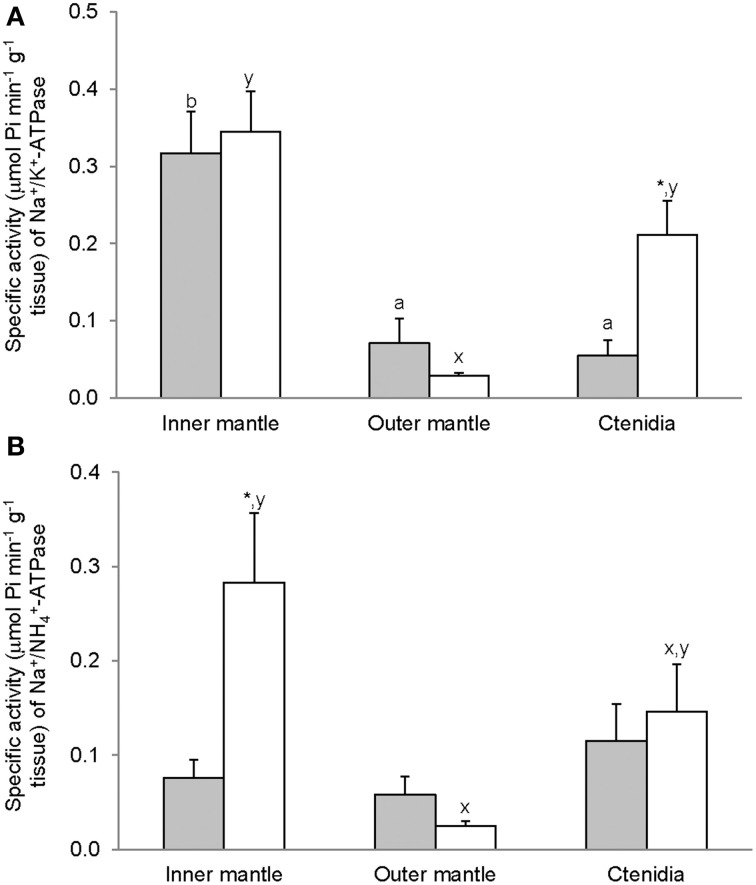
**Mass-specific activity (μmol Pi min^−1^ g^−1^ tissue) of ouabain-sensitive Na^+^/K^+^-ATPase (NKA), activated by **(A)** Na^+^/K^+^ or **(B)** Na^+^/NH^+^_4_, from the inner mantle, the outer mantle and ctenidia of *Tridacna squamosa* exposed to 12 h of darkness (control; closed bar) or 12 h of light (open bar)**. Values represent means + S.E.M. (*N* = 4). Means not sharing the same letter (a, b for darkness, or x, y for light) are significantly different from each other (*P* < 0.05). ^*^Significantly different from the control value (*P* < 0.05).

**Figure 3:**
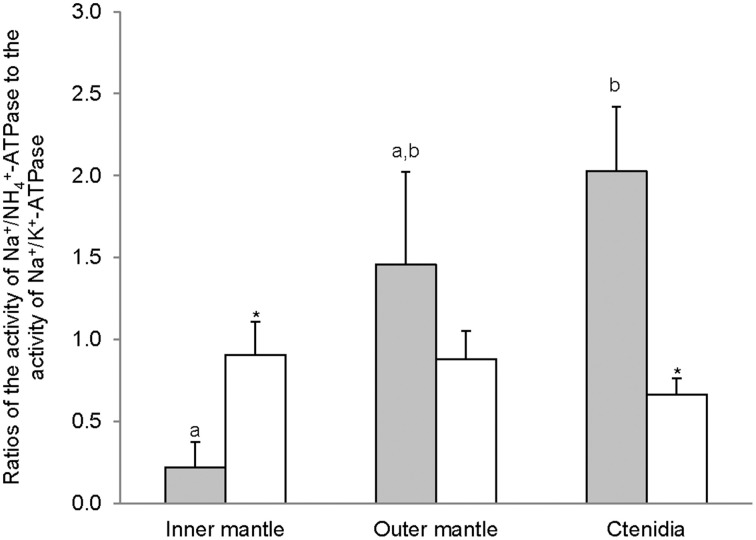
**The effectiveness of NH^+^_4_ substituting for K^+^ to activate Na^+^/K^+^-ATPase (NKA), expressed as the activity ratio of Na^+^/NH^+^_4_-activated-NKA to Na^+^/K^+^-activated-NKA, from the inner mantle, the outer mantle, and ctenidia of *Tridacna squamosa* exposed to 12 h of darkness (control; closed bar) or 12 h of light (open bar)**. Values represent means + S.E.M. (*N* = 4). Means not sharing the same letter (a, b) are significantly different from each other (*P* < 0.05). ^*^Significantly different from the control value (*P* < 0.05).

For *T. squamosa* exposed to 12 h of darkness, the inner mantle had significantly higher activity of Na^+^/K^+^-activated-NKA than the outer mantle and ctenidia (Figure 2A). Exposure to light for 12 h led to a significant increase in the Na^+^/K^+^-activated-NKA activity from the ctenidia, but not from the inner mantle and outer mantle (Figure 2A). NH^+^_4_ could also activate NKA from the inner mantle, the outer mantle and ctenidia, and the activity ratio of Na^+^/NH^+^_4_-activated-NKA to Na^+^/K^+^-activated-NKA was the lowest in the inner mantle of giant clams exposed to darkness for 12 h (Figure 3). There was a significant (3-fold) increase in the activity of Na^+^/NH^+^_4_-activated-NKA from the inner mantle of *T. squamosa* exposed to light for 12 h (Figure 2B), resulting in a significant increase in the activity ratio of Na^+^/NH^+^_4_-activated-NKA to Na^+^/K^+^-activated-NKA therein (Figure 3). Furthermore, 12 h of light exposure led to a significant decrease in the activity ratio of Na^+^/NH^+^_4_-activated-NKA to Na^+^/K^+^-activated-NKA in ctenidia of *T. squamosa* (Figure 3).

### Activities of oligomycin-sensitive H^+^/K^+^-activated-HKA and H^+^/NH^+^_4_-activated-HKA

**Figure 4:**
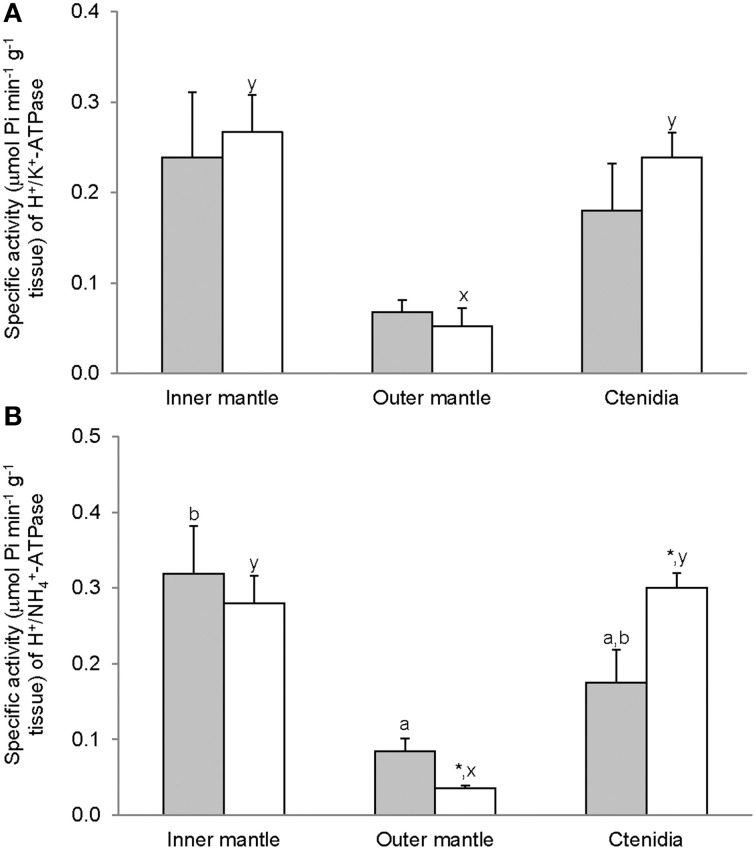
**Mass-specific activity (μmol Pi min^−1^ g^−1^ tissue) of oligomycin-sensitive H^+^/K^+^-ATPase (HKA), activated by **(A)** H^+^/K^+^ or **(B)** H^+^/NH^+^_4_, from the inner mantle, the outer mantle, and ctenidia of *Tridacna squamosa* exposed to 12 h of darkness (control; closed bar) or 12 h of light (open bar)**. Values represent means + S.E.M. (*N* = 4). Means not sharing the same letter (a, b for darkness, or x, y for light) are significantly different from each other (*P* < 0.05). ^*^Significantly different from the control value (*P* < 0.05).

**Figure 5:**
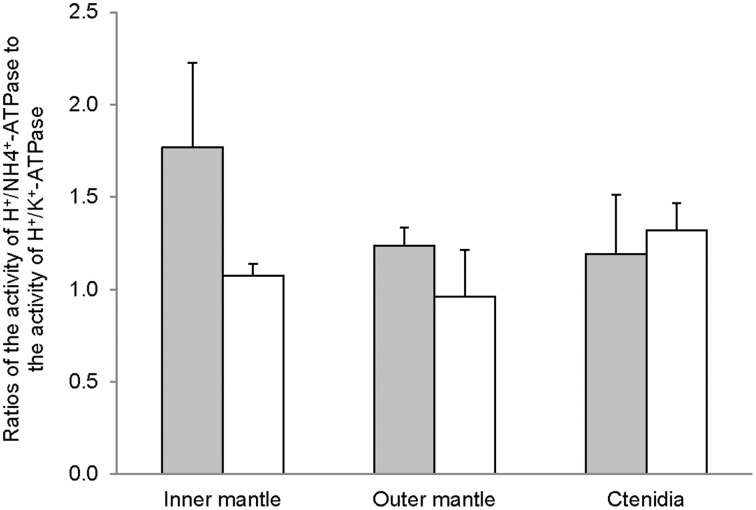
**The effectiveness of NH^+^_4_ substituting for K^+^ to activate H^+^/K^+^-ATPase (HKA), expressed as the activity ratio of H^+^/NH^+^_4_-activated HKA to H^+^/K^+^-activated-HKA, from the inner mantle, the outer mantle, and ctenidia of *Tridacna squamosa* exposed to 12 h of darkness (control; closed bar) or 12 h of light (open bar)**. Values represent means + S.E.M. (*N* = 4).

The H^+^/K^+^-activated-HKA activities from the inner mantle, outer mantle and ctenidia of *T. squamosa* were unaffected by light exposure (Figure 4A). For giant clams exposed to light for 12 h, the outer mantle had the lowest H^+^/K^+^-activated-HKA activity as compared with the inner mantle and ctenidia (Figure 4A). HKA from the inner mantle, the outer mantle, and ctenidia of *T. squamosa* could also be activated by NH^+^_4_ (Figure 4B). Exposure to light for 12 h did not affect the H^+^/NH_4^+^-activated-HKA_ activity from the inner mantle, but led to a significant decrease and a significant increase in H^+^/NH^+^_4_-activated-HKA activity from the outer mantle and ctenidia, respectively (Figure 4B). Overall, 12 h of light exposure had no significant effect on the activity ratio of H^+^/NH^+^_4_-activated HKA to H^+^/K^+^-activated HKA in the three tissues/organ of *T. squamosa* (Figure 5).

### Activities of bafilomycin-sensitive-VATPase and NEM-sensitive-VATPase

**Figure 6:**
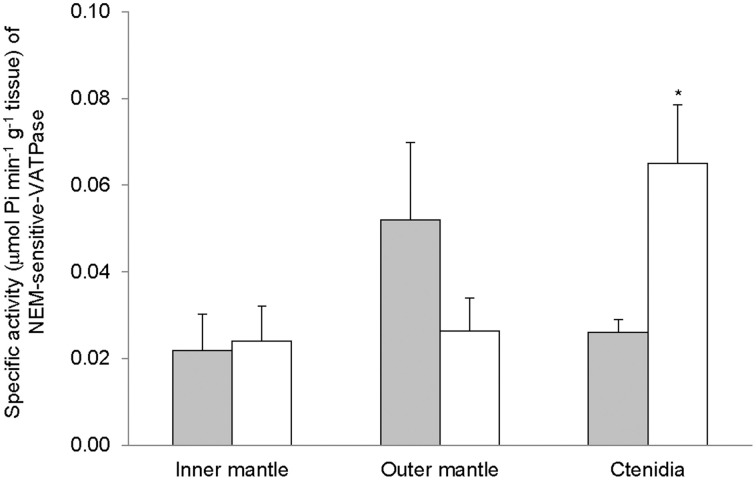
**Mass-specific activity (μmol Pi min^−1^ g^−1^ tissue) of N-ethylmaleimide (NEM)-sensitive V-type H^+^-ATPase (VATPase) from the inner mantle, the outer mantle, and ctenidia of *Tridacna squamosa* exposed to 12 h of darkness (control; closed bar) or 12 h of light (open bar)**. Values represent means + S.E.M. (*N* = 4). ^*^Significantly different from the control (*P* < 0.05).

Despite the high concentration of bafilomycin (30 μM) adopted in this study, bafilomycin-sensitive-VATPase activity was undetectable from the inner mantle, outer mantle, and ctenidia of *T. squamosa* exposed to 12 h of light or 12 h of darkness. To demonstrate that the assay system was working for bafilomycin-sensitive-VATPase, we concurrently examined the gills of the teleost, *Anabas testudineus* (Ip et al., 2012b), and obtained positive results. However, all three tissues/organ expressed NEM-sensitive-VATPase activity. The NEM-sensitive-VATPase activity from the inner mantle was unaffected by light exposure (Figure 6). While light exposure appeared to lower the NEM-sensitive-VATPase activity from the outer mantle, the difference observed was not statistically significant perhaps due to the small sampling size. By contrast, there was a significant increase in activities of NEM-sensitive-VATPase in ctenidia of giant clams exposed to 12 of light (Figure 6).

### Activities of GS, GA, and GDH

**Figure 7:**
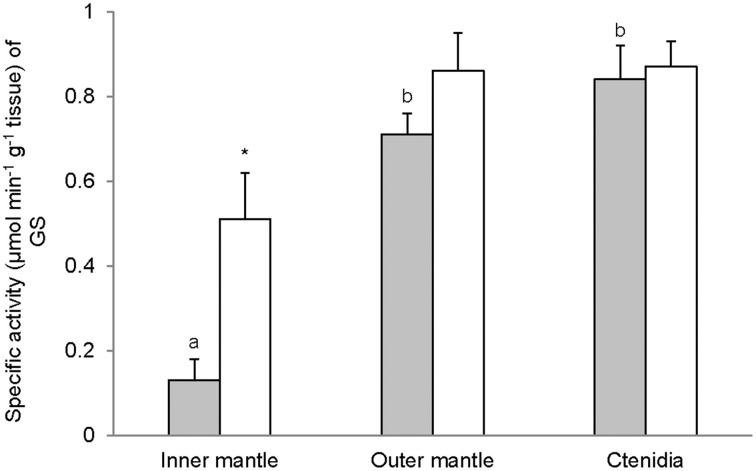
**Mass-specific activity (μmol Pi min^−1^ g^−1^ tissue) of glutamine synthetase (GS) from the inner mantle, the outer mantle, and ctenidia of *Tridacna squamosa* exposed to 12 h of darkness (control; closed bar) or 12 h of light (open bar)**. Values represent means + S.E.M. (*N* = 4). Means not sharing the same letter (a, b) are significantly different from each other (*P* < 0.05). ^*^Significantly different from the control (*P* < 0.05).

**Figure 8:**
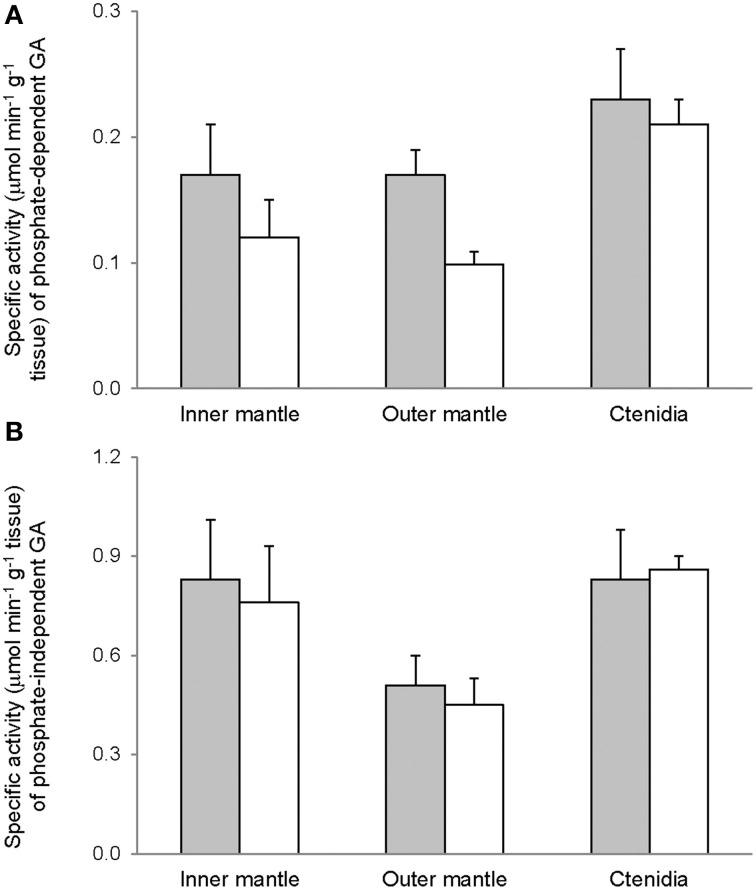
**Mass-specific activity (μmol Pi min^−1^ g^−1^ tissue + S.E.M.; *N* = 4) of **(A)** phosphate dependent glutaminase (GA) **(B)** phosphate independent GA from the inner mantle, the outer mantle, and ctenidia of *Tridacna squamosa* exposed to 12 h of darkness (control; closed bar) or 12 h of light (open bar)**. Values represent means + S.E.M. (*N* = 4).

**Figure 9:**
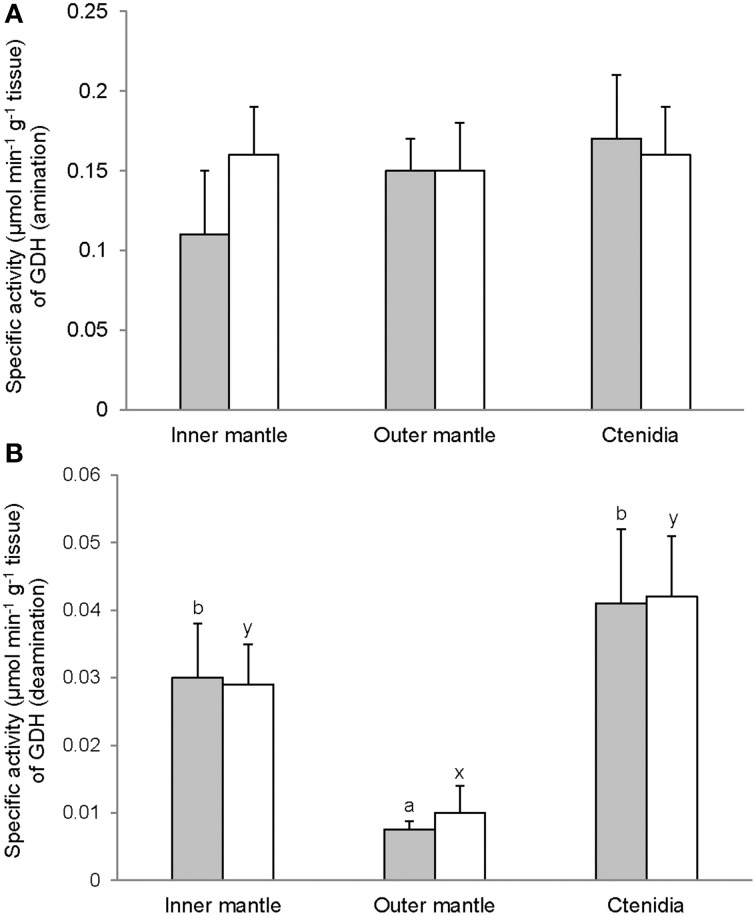
**Mass-specific activity (μmol Pi min^−1^ g^−1^ tissue + S.E.M.; *N* = 4) of glutamate dehydrogenase (GDH) in **(A)** the amination direction and **(B)** the deamination direction from the inner mantle, the outer mantle, and ctenidia of *Tridacna squamosa* exposed to 12 h of darkness (control; closed bar) or 12 h of light (open bar)**. Values represent means + S.E.M. (*N* = 4). Means not sharing the same letter (a, b for darkness, or x, y for light) are significantly different from each other (*P* < 0.05).

Activities of GS (Figure 7), GA (Figure 8), and GDH (Figure 9) were detectable in the inner mantle, outer mantle, and ctenidia of *T. squamosa*. After 12 h of exposure to light, the transferase activity of GS increased 4-fold in the inner mantle, but remained unchanged in the outer mantle and ctenidia (Figure 7). Light exposure had no significant effects on the phosphate-dependent (Figure 8A) and phosphate-independent (Figure 8B) GA activities from the inner mantle, outer mantle, and ctenidia. Of note, light exposure might lead to a decrease in the phosphate-dependent GA activity from the outer mantle (Figure 8A), but a greater number of sampling is needed to confirm the statistical significance. Similarly, the aminating (Figure 9A) and deaminating (Figure 9B) activities of GDH from the inner mantle, the outer mantle, and ctenidia of *T. squamosa* were unaffected by light.

## Discussion

After 12 h of exposure to light, there were significant increases in activities of Na^+^/NH^+^_4_-activated-NKA and GS from the inner mantle of *T. squamosa*. The extrapallial fluid, being in direct contact with the inner surface of the half-shell, is the medium through which calcification occurs (Nair and Robinson, 1998; Moura et al., 2000). On the other hand, the shell-facing epithelium of the inner mantle, being adjacent to the extrapallial fluid, is the site for the transport of various ions involved in calcification (Petit et al., 1980). Therefore, our results indicate for the first time that light-enhanced calcification in *T. squamosa* was attributable, at least in part, to the light-induced Na^+^/NH^+^_4_-activated-NKA and GS activities in the inner mantle. Notably, the light-induced changes in the inner mantle could not be a result of changes intrinsic to the symbiotic zooxanthellae. Despite having 5-fold more chlorophyll than the inner mantle, the outer mantle, which is not in direct contact with the extrapallial fluid, did not exhibit similar light-induced changes in transporter/enzyme activities. In addition, our result indicate for the first time that light induced significant increases in activities of Na^+^/K^+^-activated-NKA and H^+^/NH^+^_4_-activated HKA from ctenidia of *T. squamosa*, alluding to possible increases in Na^+^ and NH^+^_4_ transport through ctenidia during light-enhanced calcification.

### Relationships between light-enhanced calcification and the supply of Ca^2+^ to and the removal of H^+^ from the extrapallial fluid

Calcium carbonate deposition requires saturating concentrations of calcium and bicarbonate ions and produces a proton according to the reaction: Ca^2+^ + HCO^−^_3_ ⇔ CaCO_3_ + H^+^ (McConnaughey, 1995). The only way in which the equilibrium is shifted toward calcification is by the removal of protons. Indeed, Ip et al. (2006) demonstrated a significant increase in the pH of the extrapallial fluid of *T. squamosa* after exposure to light. This light-induced increase in pH would increase the supersaturation of aragonite and result in a more rapid precipitation of CaCO_3_ (Al-Horani et al., 2003). An increased rate of CaCO_3_ deposition implies that there must be an increase in the rate of Ca^2+^ supply to the extrapallial fluid during light-enhanced calcification. For *T. squamosa* exposed to light for 12 h, the activity of Ca^2+^-ATPase from the inner mantle remained comparable to that of the control exposed to 12 h of darkness, indicating that the maximal rate of Ca^2+^ transport catalyzed by Ca^2+^-ATPase could sustain the increased calcification rate induced by light. It is therefore logical to conclude that Ca^2+^-ATPase was not the rate-limiting step in light-enhanced calcification in *T. squamosa*.

The deposition and resorption of CaCO_3_ by epithelial cell layers, as in the shell-facing epithelium of the inner mantle of *T. squamosa*, require the transepithelial transport of H^+^ away from the site of calcification. In general, H^+^ can be transported by VATPase, HKA or NHE. However, all these three transporters do not facilitate H^+^ removal from extracellular fluid, as they catalyze an efflux of H^+^ from the epithelial cell. Whether the efflux of H^+^ is directed toward or away from the calcification site is dependent on the localization of the transporter in the epithelium, i.e., apical (away from the blood) or basolateral (adjacent to the blood). Transepithelial H^+^ transport is involved in calcification and decalcification in bones of vertebrates (Blair et al., 1989) and shells of molluscs and crustaceans (Cameron, 1989). Ziegler et al. (2004) used the sternal epithelium of the terrestrial isopod (*Porcellio scaber*) as a model to study epithelial H^+^ transport during CaCO_3_ formation and degradation. They reported that VATPase was expressed in the apical membrane, producing an efflux of H^+^ from the epithelial cell to the site of calcification during decalcification. By contrast, during CaCO_3_ deposition, the VATPase was expressed in the basolateral membrane of the epithelial cell, transporting H^+^ from the epithelial cell to the hemolymph (Ziegler et al., 2004). We detected NEM-sensitive-VATPase activity from the inner mantle of *T. squamosa*, but it was unaffected by 12 h of light exposure. At present, how H^+^ was transported from the site of calcification into the epithelial cell through its apical membrane remained as an enigma. For the scleractinian coral *Galaxea fascicularis*, it has been suggested that the uptake of H^+^ and efflux of Ca^2+^ at the apical membrane of the epithelial cell were coupled through the apical Ca^2+^-ATPase (Al-Horani et al., 2003), as Ca^2+^-ATPase exchanges intracellular Ca^2+^ for extracellular H^+^ (Niggli et al., 1982). While a similar process might occur in *T. squamosa* as its inner mantle exhibited Ca^2+^-ATPase activity, the possibility of H^+^ being taken up as NH^+^_4_ through some apical transporters cannot be ignored as Ip et al. (2006) demonstrated that light induced simultaneously an increase in the pH and a decrease in the ammonia concentration in its extrapallial fluid.

### Light enhanced the ability of NKA to transport NH^+^_4_ in the inner mantle

Ip et al. (2006) estimated the P_NH3_ and NH^+^_4_ concentration in the extrapallial fluid of *T. squamosa*, in light or in darkness, as 5–10 mmHg and 0.018–0.037 mmol l^−1^, respectively. For the inner mantle, the respective estimated values were 21–32 mmHg and 0.52–0.82 mmol l^−1^. Evidently, both P_NH3_ and NH^+^_4_ concentration gradients directed the passive movement of NH_3_ and NH^+^_4_ from the inner mantle to the extrapallial fluid. Hence, Ip et al. (2006) concluded that ammonia concentrations in these two compartments were displaced far from equilibrium, although the active mechanisms involved remained unknown. Results from this study reveal for the first time that the light-inducible Na^+^/NH^+^_4_-activated-NKA activity in the inner mantle could be one of the processes involved in maintaining a low total ammonia concentration in the extrapallial fluid of *T. squamosa*.

NKA is a member of the P-type ATPases, responsible for the active transport of 3 Na^+^ out of and 2 K^+^ into the cell, fuelled by the hydrolysis of ATP. It is essential for cell functions which include maintaining osmotic balance and membrane potential, and driving the secondary active transport of molecules such as glucose and amino acids (Therien and Blostein, 2000). NKA contains 2 major subunits, α and β, and functions as a αβ heterodimer. The NKA α-subunit is a large (110–120 kDa) protein that contains all the functional sites and is responsible for the catalytic functioning of the enzyme (Blanco and Mercer, 1998). NH^+^_4_ can replace K^+^ to activate NKA as they have similar hydration radius (Mallery, 1983), and NKA has been proposed to be involved in active NH^+^_4_ transport or excretion in the inner mitochondrial membrane (Campbell, 1991), the gills of shrimp (Furriel et al., 2004), the gills of crabs (Towle and Holleland, 1987; Weihrauch et al., 1999, 2002; Masui et al., 2002, 2009; Gonçalves et al., 2006; Garçon et al., 2007), and certain regions of the mammalian kidney tubule (Good and Knepper, 1985; Knepper et al., 1989; Wall, 1997). In nearly all epithelial cell types, NKA is localized basolaterally (Bystriansky and Kaplan, 2007), with the exception of the choroid plexus (Marrs et al., 1993) and the retinal pigment epithelium (Gundersen et al., 1991) whereby NKA is found along the apical surface.

Light apparently induced an increase in the effectiveness of NH^+^_4_ in substitution for K^+^ to activate NKA from the inner mantle, but not the outer mantle and ctenidia, of *T. squamosa*. It is possible that the NKA α-subunit isoform expressed in the inner mantle could be different from those expressed in the outer mantle and ctenidia. Particularly, after 12 h of exposure to light, the activity ratio of Na^+^/NH^+^_4_-activated-NKA to Na^+^/K^+^-activated-NKA for the inner mantle increased 4-fold as compared with the control exposed to 12 h of darkness. Therefore, these results land support to the proposition that H^+^ generated in the extrapallial fluid through light-enhanced calcification could be actively transported as NH^+^_4_ through a light inducible type of Na^+^/NH^+^_4_-activated-NKA into the inner mantle of *T. squamosa*. They also explain why the pH and the ammonia concentration of the extrapallial fluid increased and decreased, respectively, in *T. squamosa* exposed to light as reported by Ip et al. (2006). However, in order to pump NH^+^_4_ away from the extrapallial fluid, this light-inducible NKA, with a α-subunit that has a low substrate specificity for K^+^ (i.e., can also bind with NH^+^_4_), must be expressed atypically in the apical membrane of the shell-facing epithelium of the inner mantle, as in the case of the choroid plexus (Marrs et al., 1993) and the retinal pigment epithelium (Gundersen et al., 1991). Of note, there is evidence that suggests a basolateral localization of NKA in the outer mantle epithelium of the freshwater bivalve, *Anodonta cygnea* (da Costa et al., 1999). Hence, for *T. squamosa*, the types of NKAα and NKAβ isoforms and their subcellular localization in the inner mantle epithelium could be different from those in the outer mantle and ctenidia. Studies should be performed in the future to characterize the NKAα and NKAβ isoforms expressed in the inner mantle of *T. squamosa*, and to develop isoform-specific antibodies for the elucidation of their subcellular localization through immunofluorescence microscopy.

### Light induced an increase in the activity of GS in the inner mantle

Glutamate holds an important cross-road position in amino acid metabolism; it can donate its amino group for amino acid synthesis (transamination) via various transaminases, or lose its amino group as NH^+^_4_ via deamination to α-ketoglutarate by GDH. GDH activities, in both amination and deamination directions, were detected in the inner mantle, outer mantle and ctenidia of *T. squamosa*. In the presence of GS, glutamate can combine with NH^+^_4_ to form glutamine (Walsh and Henry, 1991). GS has been detected in host tissues from a number of alga-invertebrate associations (Rees, 1986; Rees et al., 1989; Yellowlees et al., 1994). Rees et al. (1994) reported that the host GS activity decreased by 80% in ctenidia and by 45% in the mantle of *Tridacna gigas* maintained in continuous darkness for 8 days. A similar phenomenon was observed in giant clams exposed to light but in the presence of elevated environmental ammonia concentrations. Thus, Rees et al. (1994) concluded that both host and symbionts were nitrogen deficient and that host GS played a role in ammonia assimilation by the host-symbiont association. Here, we demonstrate for the first time that light induced a ~4-fold increase in the activity of GS in the inner mantle, but not the outer mantle and ctenidia, of *T. squamosa*. As the inner mantle contained substantially less zooxanthallae than the outer mantle, it can be deduced that light has induced an increase in GS activity in the host tissues. These results corroborate the findings of Ip et al. (2006) that the inner mantle of *T. squamosa* exposed to 12 h of light contained more glutamine (2 mmol g^−1^) than that of giant clams exposed to 12 h of darkness (0.63 mmol g^−1^). When taken together, they are in support of the proposition that light induces an increase in the transport of NH^+^_4_ from the extrapallial fluid to the inner mantle, where a portion of NH^+^_4_ could be converted to glutamine. As the inner mantle also possessed GA activity, some of the glutamine formed could probably be catabolized into glutamate and NH_3_ (Walsh and Henry, 1991), although the compartmentalization of GS and GA in the inner mantle of *T. squamosa* is has not been elucidated. NH_3_ released through the glutamate-glutamine cycle could then diffuse down the P_NH3_ gradient (Ip et al., 2006) through a putative ammonia channel from the inner mantle epithelium to the extrapallial fluid to pick up more H^+^ generated during light-enhanced calcification.

### Relative high activities of H^+^/K^+^-activated-HKA and H^+^/NH^+^_4_-activated-HKA in the inner mantle and ctenidia

The active transport of K^+^ in exchange for H^+^ via two forms of HKA, gastric and non-gastric, has been reported in humans (Swarts et al., 2005, 2007; Siddaraju and Dharmesh, 2007). Furthermore, when NH^+^_4_ is used instead of K^+^ as a substrate, the oligomycin-sensitive non-gastric form of HKA from rat exhibits a higher maximum enzyme activity (Swarts et al., 2007). Indeed, our results revealed for the first time that the inner mantle, outer mantle and ctenidia of *T. squamosa* exhibited oligomycin-sensitive non-gastric H^+^/K^+^-activated-HKA and H^+^/NH^+^_4_-activated-HKA activities. Notably, the H^+^/NH^+^_4_-activated-HKA activities were comparable between the inner mantle and ctenidia, and they were significantly higher than that from the outer mantle. Unlike the Na^+^/NH^+^_4_-activated NKA activity, the H^+^/NH^+^_4_-activated HKA activity from the inner mantle was unaffected by light exposure. At present, no information is available on the localization of HKA in the inner mantle of *T. squamosa*, and its elucidation awaits future studies. However, it is unlikely that HKA would be expressed in the apical membrane of the shell-facing epithelium of the inner mantle, because pumping H^+^ from the epithelial cells into the extrapallial fluid would enhance decalcification. If it was localized atypically to the basolateral membrane of the shell-facing inner mantle epithelium, it could be involved in pumping H^+^, which could be released intracellularly through the deprotonation of NH^+^_4_ to NH_3_, from the mantle epithelial cells to the hemolymph. Light exposure might lead to a decrease in the activity ratio of H^+^/NH^+^_4_-activated HKA to H^+^/K^+^-activated HKA in the inner mantle of *T. squamosa* (Figure 5), but a greater number of sampling is needed to confirm the statistical significance.

Ctenidia of *T. squamosa* exhibited H^+^/NH^+^_4_-activated HKA activity, which increased significantly after 12 h of light exposure. Zooxanthellae symbionts are known to be nitrogen-limited, as reflected by their ability to uptake ammonia and nitrate (Wilkerson and Trench, 1986), increased photosynthesis with additions of ammonia (Summons et al., 1986), and increased growth rates of the host in responses to increases in dissolved inorganic nitrogen (Hastie and Heslinga, 1988; Onate and Naguit, 1989; Hastie et al., 1992). As ammonia present in the tridacnid host is freely available for assimilation and recycling by the zooxanthellae (Hawkins and Klumpp, 1995), it would be advantageous for the animal host to acquire the ability to actively absorb NH^+^_4_ from the external medium through its ctenidia. Indeed, it has been established that *T. squamosa* can actively absorb ammonia from the environment (Fitt et al., 1993) but there is a dearth of information on the mechanisms involved. Our results indicate for the first time that active ammonia absorption in *T. squamosa* might involve HKA, considering that it could be expressed typically in the apical membrane where it catalyzes the extrusion of H^+^ and intake of K^+^/NH^+^_4_. They offer an explanation to why tridacnids would increase their growth rates when dissolved inorganic nitrogen is added to the external medium (Hastie and Heslinga, 1988; Onate and Naguit, 1989; Hastie et al., 1992). They may also explain why other zoxanthellae-invertebrate associations excrete little or no ammonia, except when incubated in darkness (Cates and McLaughlin, 1976; Muscatine and D'Elia, 1978; Wilkerson and Muscatine, 1984; Szmant and Gassman, 1990).

### Light enhanced activities of NEM-sensitive-VATPase, Na^+^/K^+^-activated-NKA and H^+^/NH^+^_4_-activated-HKA in ctenidia

Ctenidia are not directly involved in light-enhanced calcification. Therefore, it was unexpected that exposure to light for 12 h would induce increases in activities of transporters in ctenidia of *T. squamosa*. Exposure to light led to a significant increase in the activity of NEM-sensitive-VATPase in ctenidia, indicating that light-enhanced calcification resulted in a perturbation of acid-base balance in the hemolymph which required increased H^+^ excretion. This corroborates the proposition that the excess H^+^ released through enhanced calcification was transported through the shell-facing inner mantle epithelium via NKA and HKA into the hemolymph. The light-induced increase in H^+^/NH^+^_4_-activated-HKA in ctenidia might indicate the needs to increase the uptake of NH^+^_4_ from the external medium during light-enhanced calcification (see Discussion above). Acting together in ctenidia, VATPase, and HKA would facilitate the excretion of the excess H^+^ generated through light-enhanced calcification. On the other hand, the increase in the activity of Na^+^/K^+^-activated-NKA in response to light might indicate a disturbance of Na^+^ homeostasis in the hemolymph during light-enhanced calcification, resulting in a need to increase the capacity of Na^+^ excretion through ctenidia. In mammals, the ability to deliver Ca^2+^ to the osteoid is critical to the osteoblast's function as a regulator of bone calcification. There are two known transmembrane proteins capable of translocating Ca^2+^ out of the osteoblast, the Na^+^/Ca^2+^ exchanger and the plasma membrane Ca^2+^-ATPase (Carafoli, 1987; Stains et al., 2002). These two transporters are known to be asymmetrically localized in the plasma membranes of osteoblasts, with the Na^+^/Ca^2+^ exchanger facing the bone surface (Akisaka et al., 1988; Watson et al., 1989). Because of its locations, the Na^+^/Ca^2+^ exchanger is poised to extrude Ca^2+^ into newly formed bone matrix. Hence, light-enhanced calcification in *T. squamosa* might also involve the secondary active transport of Ca^2+^ from the shell-facing inner mantle epithelial cells to the extrapallial fluid through an apical Na^+^/Ca^2+^ exchanger. If there was a transepithelial flux of Na^+^ from the extrapallial fluid to the hemolymph, there could be a need to excrete the excess Na^+^ in the hemolymph through ctenidia. Hence, it would be essential to examine the effects of light exposure on Na^+^/Ca^2+^ exchangers in the inner mantle of *T. squamosa* in future studies.

### Perspective

The finding of light being able to induce changes in activities of some transporters/enzymes involved in light-enhanced calcification in the inner mantle of *T. squamosa* is novel. To survive in an ever-changing environment, animals are equipped with mechanisms to sense environmental changes in O_2_ (Tattoli et al., 2008), pH (Monastyrskaya et al., 2008), temperature (Montell and Caterina, 2007) salinity (Berg and Ferraris, 2008) and electrical potential (Benzanila, 2008) in order to elicit appropriate physiological responses. Light-specific sensors usually comprise pigments that can be oxidized by light (Helten et al., 2007), and animal tissues without pigments are usually not light-responsive. As opsin, a photoreceptor protein, and eye-spots comprising crystalline clusters of uric acid have been found in *Symbiodinium* (Yamashita et al., 2009), it is possible that zooxanthellae living in the extensible outer mantle of *T. squamosa* can act as light-sensing elements for the tridacnid host. We hypothesize that light may induce the zooxanthellae to produce some signaling molecules, which, upon release to the animal tissue, activate a cascade of transcriptional, translational and post-translational events in the inner mantle, leading to increases in activities of transporters/enzymes essential to light-enhanced calcification. Indeed, preliminary results obtained through a differential transcriptomic analysis of the inner mantles of *T. squamosa* exposed to light vs. darkness revealed that light exposure led to increases in mRNA expression of a certain *NKAα* isoform and *GS*, alongside with many other transports and channels (e.g., *N*a^+^/Ca^2+^ *exchanger* and *Rhesus glycoproteins*) which could also be involved in the light-enhanced calcification process (Ip, unpublished results). Hence, it is highly probable that the animal host could benefit from the symbiotic zooxanthellae acting as “light-sensing” elements, in addition to the removal of CO_2_ and phosphate and the donation of photosynthates and organic matrix precursors by zooxanthellae as proposed previously to explain light-enhanced calcification.

### Conflict of interest statement

The authors declare that the research was conducted in the absence of any commercial or financial relationships that could be construed as a potential conflict of interest.
